# Double deficiency of toll-like receptors 2 and 4 alters long-term neurological sequelae in mice cured of pneumococcal meningitis

**DOI:** 10.1038/s41598-019-52212-7

**Published:** 2019-11-07

**Authors:** Lay Khoon Too, Belinda Yau, Alan G. Baxter, Iain S. McGregor, Nicholas H. Hunt

**Affiliations:** 1 The University of Sydney, Molecular Immunopathology Unit, Bosch Institute and School of Medical Sciences, University of Sydney, Sydney, New South Wales 2006 Australia; 2 Comparative Genomics Centre, James Cook University, Townsville, Queensland 4811 Australia; 3 School of Psychology, University of Sydney, Sydney, New South Wales 2006 Australia

**Keywords:** Infection, Neuroimmunology, Animal behaviour, Meningitis

## Abstract

Toll-like receptor (TLR) 2 and 4 signalling pathways are central to the body’s defence against invading pathogens during pneumococcal meningitis. Whereas several studies support their importance in innate immunity, thereby preventing host mortality, any role in protecting neurological function during meningeal infection is ill-understood. Here we investigated both the acute immunological reaction and the long-term neurobehavioural consequences of experimental pneumococcal meningitis in mice lacking both TLR2 and TLR4. The absence of these TLRs significantly impaired survival in mice inoculated intracerebroventricularly with *Streptococcus pneumoniae*. During the acute phase of infection, TLR2/4-deficient mice had lower cerebrospinal fluid concentrations of interleukin-1β, and higher interferon-γ, than their wild-type counterparts. After antibiotic cure, TLR2/4 double deficiency was associated with aggravation of behavioural impairment in mice, as shown by diurnal hypolocomotion throughout the adaptation phases in the Intellicage of TLR-deficient mice compared to their wild-type counterparts. While TLR2/4 double deficiency did not affect the cognitive ability of mice in a patrolling task, it aggravated the impairment of cognitive flexibility. We conclude that TLR2 and TLR4 are central to regulating the host inflammatory response in pneumococcal meningitis, which may mediate diverse compensatory mechanisms that protect the host not only against mortality but also long-term neurological complications.

## Introduction

Invasive infection of the central nervous system (CNS) by *Streptococcus pneumoniae* often provokes a suppurative inflammation in the arachnoid, subarachnoid space and pia mater that is known as pneumococcal meningitis. Even though the infection is curable by antibiotics and immunoprophylaxis is not uncommon, the case-fatality rate of pneumococcal meningitis remains unacceptably high in developing countries^1^. Neurological and systemic complications secondary to pneumococcal meningitis are known to contribute to deaths^2^. Pneumococcal meningitis remains a medical emergency that, without accurate diagnosis and prompt treatment, causes acute mortality in patients or, in survivors, long-lasting neuropsychological sequelae that include hearing impairment, visual deficits, mental distress, cognitive impairments and epileptic seizures^1,3^.

Harmless inhabitation by *S*. *pneumoniae* exclusively in the nasopharynx occurs in more than half of the population, especially in young children^4^. Under healthy conditions, pneumococci are barred from entering the circulation by natural protective barriers, such as respiratory mucus, lysozyme and pneumococcal IgA1 protease. When asymptomatic carriers, or persons in close contact with carriers, suffer from compromised immunity, pneumococcal invasion into the circulatory system can occur; if left unresolved by peripheral immune cells, the bacteria may subsequently cross the blood-brain barrier (BBB), entering the brain parenchyma and cerebrospinal fluid (CSF). The presence of pneumococci in the CNS is recognised by the pattern recognition receptors (PRRs) expressed in innate immune cells, such as microglia and astrocytes. The key PRRs include Toll-like receptor (TLR) 2, which is activated by lipotechoic acid^5–7^, TLR4 (activated by pneumolysin)^8^, TLR9 (activated by pneumococcal CpG-DNA)^9^, as well as nucleotide-binding oligomerisation domain-like receptors (NLRs) that sense various endogenous and exogenous stimuli^10^.

Studies in mice with targeted deletion of TLR receptors have shown the importance of both TLR2 and TLR4 in driving the pathogenesis of pneumococcal meningitis, in that the blockade of TLR2 and/or TLR2/4 signalling resulted in impaired host bacterial clearance, aggravated clinical signs and graver neurological complications^11–14^. Genetic deletion of the TLR downstream effector, myeloid differentiation primary response 88 (MyD88) protein, interferes with interleukin (IL)-1 and IL-18 signalling^15^ and causes severe deficits in immune responses^16,17^, as well as hearing impairment^18^, in experimental pneumococcal meningitis. Together, these studies suggest a link between host bacterial clearance and disease severity due to a dysregulated host inflammatory response in mice with disrupted TLR2/4 signalling. Consistent with this, single nucleotide polymorphisms (SNP) of genes responsible for bacterial sensing and their associated downstream signalling have been implicated in the prognosis of, and susceptibility to, bacterial infections^19,20^. While TLR2 + 2477 G/A polymorphism is linked to heightened risk of pneumococcal meningitis^21^, pneumococcus-infected persons with certain SNP in *TLR4* are at increased risk of developing invasive diseases^22^. Moreover, children or patients with certain SNPs in the IL-1 receptor-associated kinase 4 (*IRAK4*) gene, the nuclear factor kappa B (NF- kB) activation genes, and *MYD88*, have been associated with increased susceptibility to invasive pneumococcal diseases, which are often recurrent^23–26^. The leukocytes of patients with defects in *IRAK4*, for example, have been shown *in vitro* to be unresponsive to lipopolysaccharide (LPS) stimulation^27^. Despite these observations, associations between TLR receptor signalling and the neurocognitive sequelae of pneumococcal meningitis in survivors have not previously been determined, and we focus on this issue in the present study.

TLRs 2 and 4 are each capable of compensating for the absence of the other molecule in the acute immune and inflammatory response during pneumococcal meningitis^11,28^. In the present study, we assessed the acute CSF cytokine profile during intracranial *S*. *pneumoniae* infection in mice deficient in both *TLR2* and *TLR4*. In parallel, we measured neurocognitive activity in infected animals with the same genetic deficiencies that had been antibiotic-cured, which is an established model of long-term neurological problems resulting from pneumococcal meningitis^29^. We hypothesised that the absence of both the TLR2 and TLR4 signalling pathways would alter the innate immune response during acute pneumococcal meningitis, in part by modulating the production of inflammatory cytokines, which in turn would impact upon the long-term neurological outcomes resulting from the disease.

## Results

### Pneumococcal meningitis is exacerbated in the absence of TLR 2 and 4 signalling

**Figure 1:**
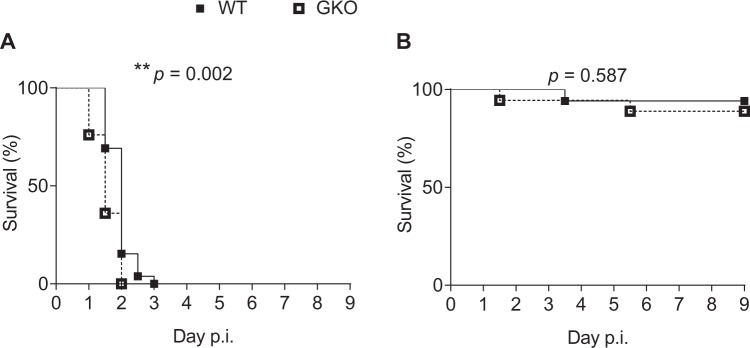
Genetic deletion of functional TLRs 2 and 4 significantly impairs survival of mice inoculated centrally with *S*. *pneumoniae*. WT = wild-type; GKO = gene knockout. The disease course of pneumococcal meningitis in WT (filled square) and GKO (unfilled square) mice was monitored up to day 9 post-inoculation (p.i., ∼5 × 10^5^ cfu *S*. *pneumoniae*/mouse) when (**A**) antibiotic ceftriaxone treatment was not administered or when (**B**) treatment was initiated at 20 h p.i. A death event was recorded when mice developed severe disease signs requiring euthanasia. Data are presented as percentage of survival at each time point, with total n = 25 WT or 26 GKO mice in three independent experiments. **p < 0.01, Log-rank test.

Without antibiotic treatment, all mice developed fatal clinical signs and were euthanased (Fig. 1A). Comparison of survival curves demonstrated increased disease severity in the GKO animals (*p* = 0.002, Log-rank (Mantel-Cox) Test). The cumulative morbidity after 1.5 days was more than twice as high in GKO compared to WT mice (64% vs 31%). On the other hand, the survival curves of WT and GKO mice were not significantly different when pneumococcal meningitis was treated with CEFT antibiotic initiated at 20 h p.i. (Fig. 1B).

### Altered inflammatory response in mice lacking functional TLR2/4 proteins during acute pneumococcal meningitis

**Figure 2:**
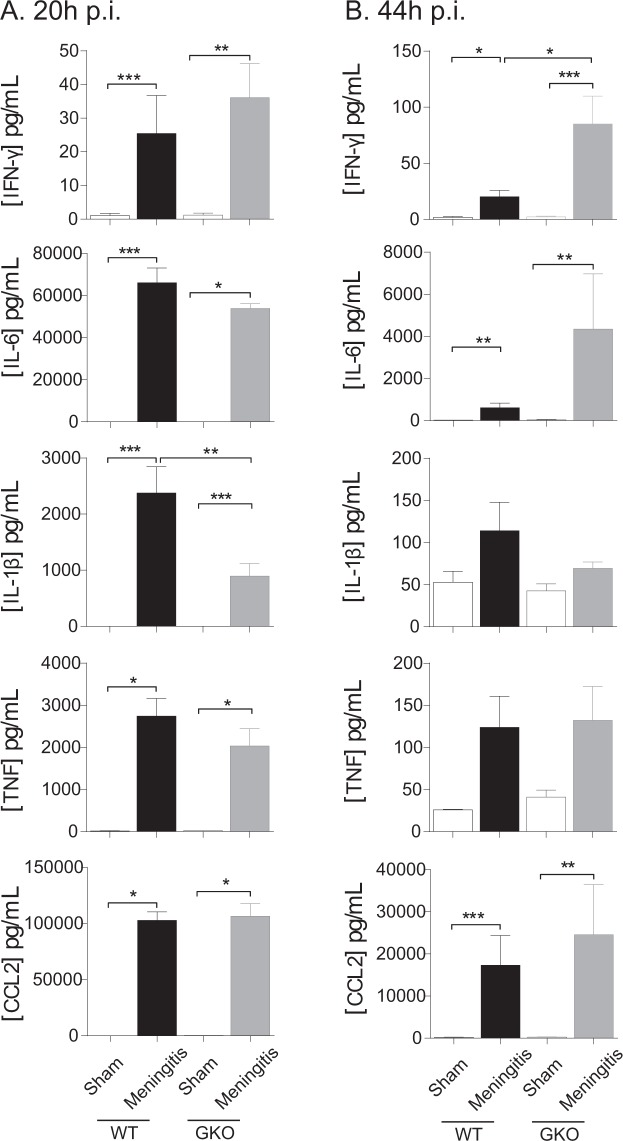
Genetic deletion of functional TLRs 2 and 4 modifies the inflammatory response in mice with pneumococcal meningitis. The CSF levels of IFN-γ, IL-6, IL-1β, TNF and CCL2 were measured at (**A**) 20 h p.i and (**B**) 24 h following the first dose of antibiotic treatment (=44 h p.i.). Antibiotic treatment commenced at 20 h p.i on all mice. WT = wild-type; GKO = gene knockout. Total n = 2–3 mice per WT or GKO sham group or n = 6–8 per WT or GKO meningitis group. *p < 0.05, **p < 0.01, ***p < 0.001, planned com*p*arisons between sham-inoculated and meningitis groups of either genotype as well as between WT and GKO meningitis groups on log-transformed data.

The concentrations of the inflammatory mediators assayed, IFN-γ, IL-6, IL-1β, TNF and CCL2, were elevated in the CSF of both WT and GKO infected mice as early as 20 h p.i. as compared to their sham-treated counterparts (Fig. 2A). The concentrations of the cytokines IFN-γ, IL-6 and TNF and the chemokine CCL2 were not significantly different between the WT and GKO meningitis animals, but a significantly lower concentration of IL-1β in the CSF was observed in the GKOs (*p* = 0.005). At 44 h p.i., the CSF levels of IFN-γ, IL-6 and CCL2 remained elevated in infected animals of both the WT and GKO genotypes (Fig. 2B). The CSF IFN-γ level was found to be significantly higher in GKOs compared to their WT counterparts (*p* = 0.020). These observations are consistent with a partial immunomodulatory role of TLRs 2 and 4 during the acute phase of pneumococcal meningitis, operating either synergistically or independently.

### IntelliCage behavioural and cognitive evaluation

As shown in our previous study^30^, C57BL/6J mice deficient in *TLR2* and *TLR4* differed from the equivalent WT mice in terms of exploratory behaviours and cognition, as measured in the IntelliCage. To account for the basal behavioural differences associated with the two genotypes, a multifactorial ANOVA of genotype by group effect was applied or a delta value of each behavioural parameter was quantified and analysed by offsetting the basal values of sham-treated animals of the relevant genotype.

#### Exploratory activities in adaptation phases

The behaviours of cage exploration, corner chamber search and drinking of a mouse within a corner chamber were assessed by measuring the frequencies of corner visits, visits with nosepokes and visits with water bottle licks, respectively, throughout the initial 5 h of FA when mice were first exposed (R1) and re-exposed (R2) to the novel IntelliCage environment in the light, followed by the dark, phases. These behaviours were also measured over the 6-day adaptation period.

**Figure 3:**
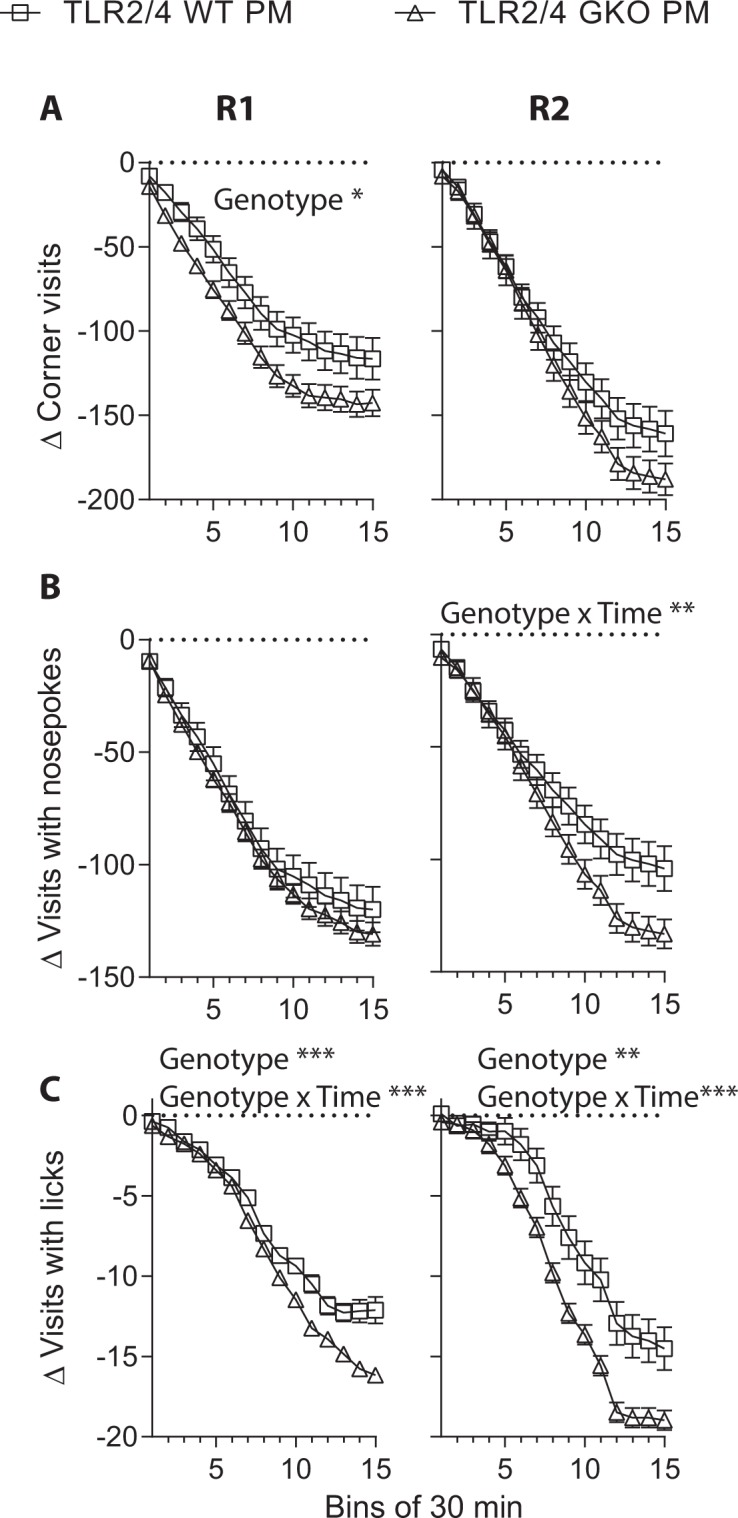
Deficiencies in TLRs 2 and 4 aggravated long-term diurnal hypolocomotion in early free adaptation in mice that survived pneumococcal meningitis due to ceftriaxone treatment (post-meningitic mice, PM). Since TLR2/4 deficiency affects exploratory behaviour in uninfected mice^30^, the corresponding delta values of visit frequency (**A**), of visits with nosepokes (**B**) and of visits with licks (**C**) are presented by offsetting the mean values of control mice. Total n = 12–14 per sham group or n = 15–17 per meningitis group. *p < 0.05, **p < 0.01, ***p < 0.001, mixed ANOVA by GLM repeated measures. Note statistical analysis on cumulative 10-min time bins collected over a 5 h period. A larger time bin (30 min vs. 10 min) is used for graphic clarity. (Abbreviations: GKO = gene knockout. WT = wild-type).

TLR2/4 deficiency aggravated post-meningitis behavioural abnormalities: The pneumococcus-infected surviving (“PM”) WT and GKO mice exhibited a significantly reduced frequency of diurnal corner visits, visits with nosepokes, and visits with licks compared to their uninfected counterparts throughout the initial 5 h of exploration in the FA paradigm during their first exposure (Suppl. Table 1, part a) and re-exposure (R2) to the IntelliCage (Suppl. Fig. 1). Analysis of delta visit frequency found a larger GKO group difference than that of WT animals in R1 (Fig. 3A: Genotype effect *F*(1, 29) = 4.79, *p* = 0.037), which diminished in R2 (Fig. 3A). On the other hand, the GKO group exhibited larger differences in visits with nosepokes and visits with licks in R1, and these became more prominent in R2 (Fig. 3B: Genotype x time, *F*(1.36, 36.67) = 7.53, *p* = 0.005; Fig. 3C: Genotype effect, *p* = 0.009; Genotype x time, *p* = 0.001). This indicated a significantly larger reduction in diurnal exploratory behaviours in the surviving GKO mice compared to their WT counterparts.

**Figure 4:**
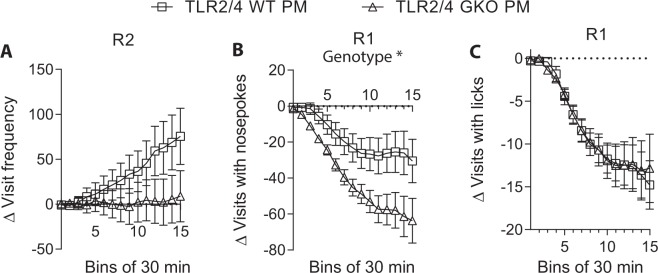
Deficiencies in TLRs 2 and 4 mildly altered nocturnal behaviours during early dark-phase free adaptation in mice that survived pneumococcal meningitis due to ceftriaxone treatment (post-meningitic mice, PM). Since TLR2/4 deficiency affects exploratory behaviour in uninfected mice^30^, the corresponding delta values of visit frequency in R2 (**A**), of visits with nosepokes in R1 (**B**) and of visits with licks in R1 (**C**) are presented by subtracting the mean values of respective sham group from individual mouse activity level. The data of nocturnal visits with nosepokes and licks from R2 and that of nocturnal corner visits in R1 are not shown due to the absence of WT or GKO group effect (refers Suppl. Fig. 2 for group effect on pre-converted data). Total n as indicated in Fig. 3. *p < 0.05, overall genotype effect as analysed by mixed ANOVA by GLM repeated measures. Note statistical analysis on cumulative 10-min time bins collected over a 5 h period. A larger time bin (30 min vs. 10 min) is used for graphic clarity. (Abbreviations: GKO = gene knockout. WT = wild-type).

Subsequent to the light cycle, exploratory behaviours throughout the first 5 h of the dark cycle were assessed. In R1, there was no significant genotype effect when the frequencies of corner visits throughout the initial 5 h of dark phase were compared between PM mice and the sham-infected animals (Suppl. Table 1, part b). In R2, WT PM mice were found to display a higher level of corner-visiting activity than their sham-infected controls (Suppl. Fig. 2A: Group *F*(1, 29) = 2.17, *p* = 0.152, group x time *F*(1.16, 33.56) = 4.23, *p* = 0.042). In contrast, a significant group effect was absent in the GKO animals. Analysis of delta visit frequency, however, showed no significant genotype effect (Fig. 4A). On the other hand, the frequencies of nocturnal visits with nosepokes or licks were significantly lower in PM than sham mice of both genotypes in R1 (Suppl. Table 1, part b) (Suppl. Fig. 2B,C, respectively). A significantly larger group difference was found in the GKO group compared to the WT group when the delta visits with nosepokes was analysed (Fig. 4B: Genotype *F*(1, 29) = 5.44, *p* = 0.027), but not when delta visits with licks was investigated (Fig. 4C). A significant group or genotype effect was however absent in R2 (data not shown).

Overall, these results showed that the absence of TLR2/4 signalling in mice exacerbated the long-term PM effect, leading to worsened diurnal behavioural hypoactivity in the PM GKO mice.

#### TLR 2/4 signalling contributes to abnormal behavioural adaptation in pneumococcal meningitis

**Figure 5:**
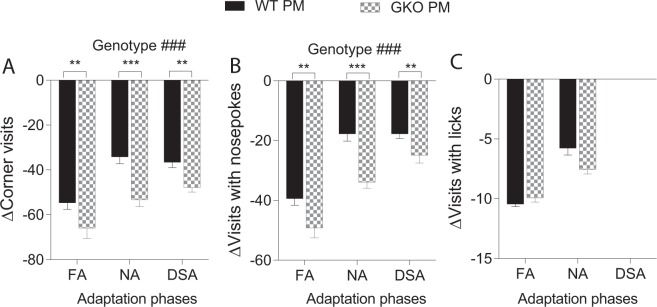
Post-meningitic (PM) mice deficient in TLR2/4 signalling displayed worsened diurnal hypolocomotion during adaptation phases in the long-term. All data are from the re-testing protocol (R2). The frequency of diurnal corner visits (**A**) in each two-day free adaptation (FA), nosepoke adaptation (NA) and drinking session adaptation (DSA) was summed. Since TLR2/4 deficiency affects exploratory behaviour in uninfected mice^30^, the corresponding delta values of corner visits (A) are presented to show genotype differences related to pneumococcal meningitis. Similarly, delta values of visits with nosepokes (**B**) and licks (**C**) from the adaptation phases are plotted. Total n as shown in Fig. 3. ^###^*p* < 0.001, overall genotype effect as analysed by mixed ANOVA by GLM repeated measures. **p < 0.01, ***p < 0.001, genotype effect as analysed by Fisher’s LSD. WT = wild-type; GKO = gene knockout.

Mixed ANOVA of exploratory behaviours throughout the adaptation phases showed that the PM mice of both WT and GKO genotypes displayed significantly reduced corner visits, visits with nosepokes and visits with licks compared to their sham-infected counterparts in both R1 (Suppl. Table 1, part c) and R2 (Suppl. Fig. 3). Further evaluation of delta values of corner visits in R1 showed a significant overall genotype effect (Fig. 5A: *F*(1, 27) = 20.41, *p* < 0.001) in R2, but not in R1, indicating a larger PM effect as a result of TLR2/4 deficiency. Similar outcomes were obtained when visits with nosepokes (Fig. 5B: Genotype *F*(1, 26) = 22.96, *p* < 0.001), but not visits with licks (Fig. 5C), were analysed.

**Figure 6:**
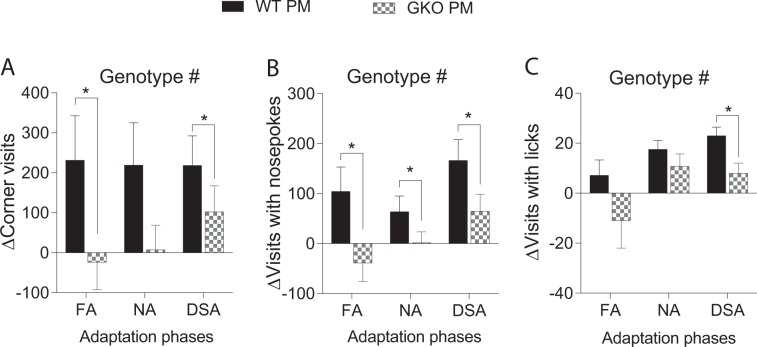
Post-meningitic (PM) mice deficient in TLR2/4 signalling displayed ameliorated nocturnal hyperactivity throughout the adaptation phases in the long-term. All data are from the re-testing protocol (R2). The frequency of nocturnal corner visits (**A**) in each two-day free adaptation (FA), nosepoke adaptation (NA) and drinking session adaptation (DSA) was summed. Since TLR2/4 deficiency affects exploratory behaviour in uninfected mice^30^, the corresponding delta value of corner visit frequency (**A**) are presented to show genotype difference related to pneumococcal meningitis. Similarly, delta frequencies of visits with nosepokes (**B**) and licks (**C**) are plotted over each adaptations phase. Total n as shown in Fig. 3. ^#^*p* < 0.05, overall genotype effect as analysed by mixed ANOVA by GLM repeated measures. *p < 0.05, genotype effect as analysed by Fisher’s LSD. WT = wild-type; GKO = gene knockout.

Over the dark cycle of adaptation phases in R1, neither a significant overall group nor genotype effect was observed on frequencies of corner visits, visits with nosepokes and visits with licks (Suppl. Table 1, part d). On the other hand, analysis of delta values collected from R2 showed a significant overall genotype effect on corner visit frequency (Fig. 6A: *F*(1, 25) = 4.31, *p* = 0.048), visits with nosepokes (Fig. 6B: *F*(1, 25) = 7.56, *p* = 0.011) and visits with licks (Fig. 6C: *F*(1, 26) = 6.02, *p* = 0.021), indicating a significantly smaller group difference between the GKO sham and PM mice.

In summary, the manifestation of diurnal hypolocomotion in mice following recovery from pneumococcal meningitis after CEFT treatment was aggravated by TLR2/4 deficiencies. In contrast, nocturnal hyperactivity was ameliorated in the absence of TLR2/4 signalling during pneumococcal meningitis. For both phenotypes, the TLR2/4-associated PM effects were more evident in R2 than in R1.

#### TLR2/4 signalling is not responsible for altering response to light stimulus in PM mice

The response of mice to different light stimuli was measured to assess their preference or avoidance behaviours. The frequency of visits to the corner showing a light stimulus was analysed using simple contrast, with the reference category set at a baseline level equivalent to the percent visits to that specific corner in the second day of DSA. A similar behavioural response to light stimuli was observed in both R1 and R2, hence only observations in R2 are presented (Suppl. Fig. 4). In R2, a significant PM effect on both WT and GKO mice was seen as a reduction in preference for a flashing RBG in PM mice compared to their sham-infected equivalents (WT: *F*(1, 24) = 26.59, *p* < 0.001; GKO: *F*(1, 22) = 7.07, *p* = 0.014). Subsequently, PM mice of either genotype manifested avoidance behaviour towards an RBG that was constantly illuminated, a phenotype that was not detected in sham-infected controls (WT: *F*(1, 24) = 57.47, *p* < 0.001; GKO: *F*(1, 22) = 24.17, *p* < 0.001). Multifactorial ANOVA of group by genotype interaction during each type of light stimulus showed no significant genotype effect, implying a similar degree of altered light response in WT PM mice and those deficient in TLR2/4 signalling, with respect to the corresponding sham controls. Together, these results indicate that the PM effect on both short- and long-term changes in mouse sensitivity to light stimuli is TLR2/4-independent.

#### The learning ability of post-meningitic mice, measured in patrolling tasks, was not affected by TLR2/4 deficiencies

Analysis of learning performance of mice subjected to the simple patrolling task in R1 by multifactorial ANOVA showed no significant difference in learning ability between WT and GKO PM mice. When mice were subsequently subjected to the complex patrolling task, we detected a similar degree of learning deficiency in WT PM mice compared to their GKO equivalents.

When they were re-tested in the same task in R2, both WT and GKO PM mice manifested impaired performance in the simple patrolling task compared to their respective sham-infected controls (Suppl. Fig. 5A: WT Group *F*(1,21) = 13.44, *p* = 0.001; GKO Group (*F*(1,18) = 43.40, *p* < 0.001). Multifactorial ANOVA showed no significant genotype effect on the performance level of mice. Additionally, the PM mice of both genotypes did not perform as well as the uninfected counterparts in the complex patrolling task, as evidenced by a significant group effect (Suppl. Fig. 5B: WT Group *F*(1,19) = 74.83, *p* < 0.001; GKO Group *F*(1,19) = 41.24, *p* < 0.001). All mice similarly reduced their visits to the non-rewarding reference corner assigned randomly to them in both simple and complex patrolling tasks, not only in first (R1, data not shown) but also second exposure (R2, Suppl. Fig. 4C,D) to the tasks. We therefore conclude that the mouse learning capacity in this task was not significantly affected by TLR2/4 deficiency.

#### Cognitive inflexibility after meningitis was exacerbated by TLR2/4 deficiency

When mice were subjected to a spatial reversal task for the first time (R1), GKO PM mice showed a significantly lowered performance compared to the sham equivalents, a phenomenon that was not observed in WT animals. The difference in performance level between GKO sham-infected and PM mice did not however contribute to a significant genotype effect as analysed by multifactorial ANOVA, implying that learning impairment was not associated with TLR2/4. When mice proceeded to a discrimination reversal task, the PM mice of both genotypes displayed suboptimal performance compared to their corresponding uninfected counterparts (WT Group *F*(1, 22) = 4.58, *p* = 0.044; GKO Group *F*(1, 20) = 25.75, *p* < 0.001, figure not shown). A significant genotype effect was not observed.

**Figure 7:**
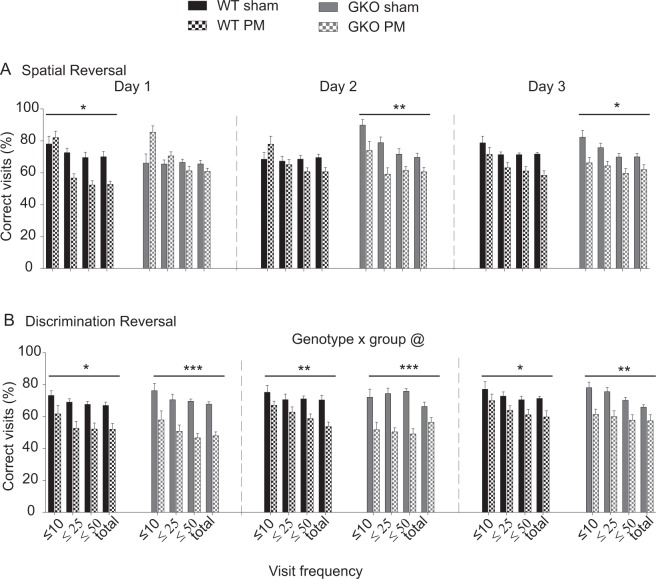
TLR2/4 signalling modulates long-term executive functioning in post-meningitic mice (PM). All data are from the re-testing protocol (R2). The percentage of correct visits was measured in (**A**) spatial and (**B**) discrimination reversal tasks. Total n as shown in Fig. 3. *p < 0.05, **p < 0.01, ***p < 0.001, group effect on each test day as analysed by Fisher’s LSD. ^@^*p* < 0.05, mixed ANOVA by GLM repeated measures. WT = wild-type; GKO = gene knockout.

When mice were re-exposed to the same sets of test paradigms in R2, GKO PM mice again showed a learning deficit in the spatial reversal task (Fig. 7A: *F*(1, 20) = 9.40, *p* = 0.006). The deficit was also manifested in WT PM mice (Fig. 7A: *F*(1, 23) = 5.63, *p* = 0.026). Multifactorial ANOVA showed that PM mice of both genotypes experienced a similar degree of impairment in the spatial reversal task. When they were re-exposed to a discrimination reversal task, WT and GKO PM mice showed a significantly lower performance level compared to their sham controls (Fig. 7B: WT Group *F*(1, 20) = 13.66, *p* = 0.001); GKO Group *F*(1, 16) = 46.79, *p* < 0.001). Notably, this deficit was more evident in GKO PM mice, where a significant genotype by group effect was detected by multifactorial ANOVA (*F*(1, 36) = 5.92, *p* = 0.020). In both spatial and discrimination reversal tasks, sham or PM mice of either genotype were able to identify the reference corner, as demonstrated by reducing their visits to below the 25% chance level in R1 (data not shown) and R2 (Suppl. Fig. 6A,B).

In summary, long-term cognitive impairment of mice in the visual discrimination task was aggravated in the absence of TLR2/4 signalling during acute pneumococcal meningitis.

## Discussion

TLR2 and TLR4, which are expressed on antigen-presenting cells, respectively sense pneumococcal lipoteichoic acids^6^ and pneumolysin^8^ and thereby trigger a host inflammatory response against pneumococcal infection. The inflammatory process leading to the bactericidal effect is believed to simultaneously cause neurological complications that can either be resolved upon recovery, such as cerebral oedema, or associated with long-term neuropsychological problems, such as hearing loss, psychological distress, and cognitive impairment^31^. The detailed pathological mechanisms underlying PM neurological sequelae remain poorly understood. Using an established mouse model, the present study demonstrates a partial role for a TLR2- and TLR4-mediated acute inflammatory response in modulating the long-term neurological outcomes seen in mice that have survived pneumococcal meningitis due to CEFT treatment.

### TLR2/4 signalling plays a partial role in the acute immune response in pneumococcal meningitis

TLR activation initiates both the MyD88 and the TIR domain-containing adaptor inducing IFN-β (TRIF) signalling pathways to drive NF-kB and activating protein-1 (AP1) nuclear translocation in inflammatory states^32^, inducing the production of proinflammatory cytokines. In the absence of CEFT treatment, TLR2/4 deficiency results in an immune system dysfunction, primarily in host cell recognition of pneumococcus microbial patterns^11^. Our present findings are congruous with those of Klein and colleagues, who similarly report lesser increases in IL-1β, but comparable concentrations of CCL2 and IL-6, within the brains of TLR2/4 double GKO mice in a pneumococcal meningitis model^11^. In addition to TLRs, the release of IL-1β from leukocytes is dependent upon another, similar microbial pattern recognition system, the NOD-like receptor (NLR) inflammasome^33^. This therefore demonstrates a non-exclusive role of TLR2 and TLR4 in mediating production of proinflammatory cytokines, in particular IL-1β, and that the loss of TLR2/4 function is not fully compensated for by NLR signalling.

Without therapeutic intervention, excessive bacterial growth correlates with increased mortality; worsened survival outcomes have been reported in TLR2 single GKO mice inoculated intracisternally or intracerebrally with *S*. *pneumoniae*, accompanied by increased bacterial load within the brain and/or blood, compared to WT controls^12,14^. Moreover, TLR2/4 double GKO mice intracisternally injected with *S*. *pneumoniae* show worsened clinical outcomes with increased bacterial load in the brain and the blood^11^. In contrast, neither impaired survival nor altered bacterial titres in the brain and blood have been reported in TLR4 single deficient mice^11^. These data together suggest that the TLR2 pathway, independent of TLR4, may regulate bacterial outgrowth within cerebral models of pneumococcal infection^14,34^. Furthermore, TLR2 deficiency leads to a decreased proinflammatory cytokine profile, including TNF and IL-1β, in a murine model of *S*. *pneumoniae* otitis, resulting in increased bacterial outgrowth, bacteraemia and death^35^. In agreement with these studies, our findings suggest that TLR2 and TLR4 collaboratively regulate the innate inflammatory response during the acute stage of pneumococcal meningitis, dysregulation of which due to the lack of TLR2/4 has contributed to the increased early mortality of TLR2/4-deficient mice. Compared to MyD88 deficiency^16,36^, which profoundly suppresses host immunity against pathogens, double deficiency in TLR2 and TLR4 only modestly affected the immune response (as measured by immune mediator levels in the CSF) and mortality due to acute pneumococcal meningitis, likely due to a compensatory mechanism by another microbial pattern recognition system. It is noteworthy that the molecular events that link bacterial burden to death remains to be established, and the early mortality of TLR2/4-deficient mice may also be attributable to neurological complication(s).

In our current report using CEFT treatment, antibiotic killing of pneumococci and prevention of bacterial outgrowth resulted in similar survival rates in WT and GKO mice. In contrast to *S*. *pneumoniae*-infected WT mice, which showed an attenuated increase in CSF IFN-γ concentration following a single dose of CEFT, IFN-γ continued to increase by 3-fold in the GKO mice (Fig. 2A,B). We previously have demonstrated a causative link between IFN-γ and detrimental outcomes in pneumococcal meningitis for acute fatality (without antibiotic)^37^ and cognitive outcomes (with antibiotic)^38^. The augmented CSF IFN-γ response observed in the present study, however, did not coincide with impaired survival in GKO mice receiving CEFT treatment since they were mostly cured. On the other hand, when antibiotic intervention is given, the presence of higher concentrations of the pro-inflammatory cytokine IFN-γ at the later stages of disease progression might participate in the ensuing pathological mechanism(s) that lead to long-term neurological problems.

### Deficiency in TLR2 and TLR4 alters behavioural and cognitive sequelae in experimental pneumococcal meningitis

TLR2, TLR4 and TLR9 single-nucleotide polymorphisms are associated with increased risks of neurological sequelae, specifically loss of hearing, in meningococcal meningitis and pneumococcal meningitis^39^. In addition, polymorphisms in other microbial pattern recognition receptor genes, such as the inflammasome NLRP1 gene, as well as CSF IL-1β concentration correlate with poor disease prognosis in pneumococcal meningitis^40^. Our results open the possibility that identifiable pathways drive neurological outcomes, at least in part: TLR2/4 regulate host immunity, in particular, so that their deficiency results in a heightened IFN-γ CSF profile post-CEFT treatment that correlates with the aggravation of long-term diurnal hypolocomotion and impairment in cognitive flexibility. In support of this, we previously showed that these behavioural and cognitive changes are not seen in IFN-γ GKO mice^38^. This indicates that TLR2/4 activation is likely an upstream regulator of IFN-γ-dependent neuropathological outcomes in pneumococcal meningitis, though other factors undoubtedly contribute, as discussed below.

As demonstrated in our previous studies^38,41^, a nocturnal hyperactive phenotype was absent in both IFN-γ and IL-18 GKO mice that were cured of pneumococcal meningitis by CEFT – a phenomenon also observed in the present TLR2/4 GKO PM mice, notwithstanding a varying pattern of diurnal locomotion (less hypoactive in IFN-γ GKO, equally hypoactive in IL-18 GKO and more hypoactive in the current TLR2/4 GKO PM mice compared to the respective WT equivalents). These mice share one common cytokine profile – reduction in CSF IL-1β. TNF, IL-6 and IL-1β are well-known markers of sickness behaviour during inflammation^42^ and their effect can potentially be long-lasting^43^, so it is likely that reduced IL-1β production due to TLR2/4 deficiency during the acute stage of pneumococcal meningitis is associated with amelioration of the hyperactive phenotype in PM mice that survived pneumococcal meningitis due to CEFT treatment. A recent study reports the protective effect of IL-1β receptor antagonist on short-term (10 days p.i.) cognitive impairment (habituation memory, novel object recognition and aversive memory) due to acute pneumococcal meningitis^44^. However, an attenuated increase in CSF IL-1β concentration in TLR2/4 GKO mice in our meningitis mouse model did not confer protection from learning impairment in mice subjected to patrolling or reversal tasks. Therefore, it is likely that the CSF IL-1β concentration in TLR2/4 GKO mice in our study reached a threshold sufficient to cause learning impairment and that an imbalanced cytokine profile leads to worsened consequences. Alternatively, these observations are consistent with cytokine-specific modulation of different cognitive subsets.

Apart from TLR2/4-mediated inflammatory cytokine expression, alternative TLR2/4-associated mechanisms may play important roles in driving the pathogenesis of long-term neurological sequelae: (1) TLR2/4 signalling regulates antibacterial mechanisms and/or leucocytosis, disruption of which leads to impaired bacterial clearance, accumulation of inflammatory bacterial debris, and a dysregulated host inflammatory response, resulting in increased early mortality and/or long-term neurological changes. This mechanism is consistent with previous studies^11–13^ conducted to explore the acute pathogenesis of pneumococcal meningitis that link CNS bacterial burden to increased disease severity/mortality in TLR2- and/or TLR4-deficient mice. In addition, Klein and colleagues^11^ have reported in TLR2/4-deficient mice with pneumococcal meningitis a 50% attenuation of the increase in CSF white cell count. Together with our previous study investigating the effect of neutrophil depletion^45^ and a retrospective population study on patient data that demonstrated a negative correlation of CSF leucocyte count with neurological complications^2^, these studies suggest the potential involvement of TLR2/4-mediated leucocytosis in driving neurological problems in our disease model. (2) TLR2/4 via multiple mechanistic pathways, including (1) or other yet-to-be-discovered neuroprotective pathways, mediate compensatory mechanisms that protect the host against brain injury during acute pneumococcal meningitis. Similar to other neurological diseases^46^, however, the exact immunomodulatory role of TLR2/4 activation in driving neuropathological events during pneumococcal meningitis remains to be elucidated.

Besides acute inflammation, TLRs have long been believed to participate in inflammatory events during chronic neurodegenerative conditions. TLR2 and TLR4 are induced by experimental cerebral ischemia^47^; mice lacking either TLR show reduced brain injury upon cerebral focal ischemia injury^47,48^. Furthermore, TLR4-deficient mice showed improved neurological behaviours following experimental stroke^49^. On the other hand, TLRs 1–8 are upregulated in the CNS of Alzheimer’s disease patients^50^, and a haplotype-tagging SNP is associated with increased risk of developing late-onset Alzheimer’s disease^51^. Similar to these diseases, we have demonstrated in a pre-clinical model a potentially important mechanistic linkage between TLR-mediated inflammation and the development of long-term neurological deficits that might be relevant in patients cured of pneumococcal meningitis by antibiotic treatment. The therapeutic implications are worthy of further investigation.

## Conclusions

In conclusion, the TLR2/4 axis plays a partial role in modulating the acute host inflammatory response during pneumococcal meningitis that may act to prevent, or limit, mortality and neurological complications to some degree. It is likely that by modulating the inflammatory response and/or other compensatory mechanisms, TLR2/4 is central to host protection during pneumococcal meningitis.

## Material and Methods

### Experimental subjects

Female C57BL/6J mice doubly deficient in TLR2 (B6.129-*Tlr2*^*tm1Aki*^/Bax) and TLR4 (B6.129-*Tlr4*^*tm1Aki*^/Bax) were interbred at James Cook University, Australia^52,53^ and the double gene knockout (GKO) mice were subsequently bred at the Medical Foundation Building Animal House, the University of Sydney, Australia. Age-matched, wild-type (WT) control mice of C57BL/6J background were purchased from the Animal Resources Centre, Perth, Australia. Mice 7 to 12 weeks of age were included in the study, with no more than 4 weeks age range in each experiment. Unless otherwise stated, WT and GKO mice were housed in groups of 3 to 6 mice in individually-vented cages (Tecniplast, Buguggiate, Italy) with *ad libitum* access to water and food in a temperature- and light-controlled conditions. All experimental procedures were approved by the University of Sydney Animal Ethics Committee and adhered to the NSW Animal Research Act (1985 – Animal Research Regulation 2010) and the 2004 NHMRC ‘Australian code of practice for the care and use of animals for scientific purposes’.

### Induction of experimental pneumococcal meningitis

Consistent with our previous studies^38,41,54^, mice were injected with *S*. *pneumoniae* serotype 3 WU2 strain (courtesy of Prof. J. Paton, University of Adelaide, Australia) into the third cerebral ventricle under light anaesthesia with inhalant isofluorane. Concomitantly, sham-infected control animals received intracerebroventricular injection of Dulbecco’s Phosphate-Buffered Saline (DPBS). Clinical symptoms indicative of successful bacterial infection, including mild lethargy and immobility with hunched posture, were apparent at 20 h post-inoculation (p.i.), at which time antibiotic treatment was initiated. Ceftriaxone (CEFT) was administered to both pneumococcus-infected and uninfected animals as previously described^38,41^. The disease course of pneumococcal meningitis was monitored daily for up to 10 days or up to a specified sampling point (eg. 44 h p.i.). Mice with severe illness indicative of irreversible pneumococcal meningitis (marked lethargy and immobility, prominent gait disturbance, delayed righting reflex, or fitting) were euthanased. Mice that survived acute pneumococcal meningitis following CEFT treatment were subjected to long-term neurological assessment using the IntelliCage^TM^ automated cognitive and behavioural screening system, and are denoted as “post-meningitic” (PM) throughout the text.

### CSF cytokine and chemokine measurement

The first CSF collection^38^ occurred at 20 h p.i. and the second at 24 h following the first administration of CEFT (i.e. 44 h p.i.). A total of 6–8 pneumococcus-infected WT and GKO and 2–3 sham-inoculated WT and GKO mice were euthanased with an overdose of inhalant isofluorane for CSF collection. The CSF concentrations of cytokines (IFN-γ, 1L-1β, IL-6 and TNF) and the monocyte chemoattractant, chemokine (C-C motif) ligand 2 (CCL2), were measured as previously described^54^ using cytometric bead array (CBA – Becton Dickinson Biosciences).

### IntelliCage^TM^ behavioural and cognitive assessment

Due to the presence of various behavioural and cognitive deficits in both experimental and clinical cases of pneumococcal meningitis, the present study used the high-throughput, multifaceted IntelliCage test system to investigate a range of potential neurological alterations in PM animals. The test components and setup have been comprehensively described and pictorially illustrated in our previous publications^38,54^. With this functionally flexible system, we previously have studied neuropsychological effects of TLR 2 and 4 deficiencies in healthy mice^30^. By including 4 conditioning operant units into a single large home cage that is approximately 5 times the size of a standard home cage, a total of up to 16 mice can be tested concurrently and continuously with a range of software-programmed test paradigms. As described before^54^, a 16-day test battery was used to quantitatively analyse behavioural and cognitive changes post-meningitis in the absence of functional TLR 2 and 4 signalling, to illuminate the role of this signalling pathway in behavioural and learning modulations in PM mice. All mice had been treated with CEFT in order to control for any potential effects of the antibiotic upon behaviour or learning^55^.

#### Pre-designed IntelliCage test paradigms

The test battery was previously described in detail^54^. It begins with initial habituation to the novel IntelliCage environment (free adaptation, FA), to explore the corner chambers (nosepoke adaptation, NA), and to drink within specified sessions (drinking session adaptation, DSA), each for 2 days. After the behaviours of mice were shaped during these adaptation phases, their light preference/avoidance behaviours were assessed over the next two days: on day 1, a 5 s flashing Red/Blue/Green (RBG) light was initiated in corner 1 upon a visit; on day 2, a constant RBG light was initiated in corner 1 at the beginning of the test day. Following this, mice were trained in patrolling and reversal tasks as briefly described below:

1. Patrolling task (PT): Prior to the PT, mice were subjected to the DSA procedure for one day in order to restore their baseline behaviours. Two PT paradigms were designed: simple and complex. To access water (reward), both types of PT required mice to patrol to another corner after they had been rewarded. The patrolling pattern was not fixed in simple PT, but in complex PT the mice had to patrol in clockwise, then anticlockwise, directions to obtain rewards. Simple PT was executed in the first 3 h of the dark cycle preset in the animal house, and complex PT in the last 3 h. In both PTs, a set of 3 “working” and 1 “reference” corners was randomly assigned to mice. While the “reference” corner was permanently closed for reward, mice patrolled among the remaining 3 “working” corners for reward in a specified manner. A “rewarding” corner was cued by a 5 s flashing RBG light visual stimulus.
2. Reversal task (RT): Mice on the first day of the RT were again exposed to the DSA paradigm so as to restore baseline behaviour. The criterion for obtaining a reward in the RT was the same as in the simple PT, which required mice to randomly seek another “rewarding” corner after they had been rewarded in a specific corner. In “spatial RT”, which was initiated during the first drinking session, the pre-assigned set of “working” and “reference” corners was switched so that the “reference” corner now moved to the next clockwise corner. In “discrimination” reversal, which occurred in the second drinking session, all corners were constantly lit at the beginning of the test and a “rewarding” corner was indicated by deactivation of the visual stimulus upon entry. The exit of a mouse from the rewarded corner re-illuminated the corner.

#### Mice

For the part of the study directed at meningitis-associated neurological sequelae, 31 WT and 29 GKO mice were used and grouped as following:

1. WT sham-infected controls (n = 14);
2. WT infected mice (n = 16);
3. GKO sham-infected controls (n = 12);
4. GKO infected mice (n = 15).

Two GKO mice and one WT mouse that were not cured of pneumococcal meningitis by CEFT treatment were euthanased and excluded from further study. Three to five mice of different genotypes that had been sham-infected or pneumococcus-infected were group housed in the same IntelliCage. Experiments were carried out on two separate mouse cohorts and the data were combined for statistical analyses. Mice were subjected twice to the 16-day test battery with a one-month interval between them, so that short- and long-term behavioural and cognitive changes could be examined. The first neurobehavioural and cognitive test cycle, which started at 10 day p.i., was termed R1, and the later one R2, which started one month after the end of R1.

### Data analysis

Statistical analysis of the data was carried out using GraphPad Prism version 5.01 for Windows (Graphpad Software, San Diego, CA, USA) and IBM SPSS Statistics version 20 (SPSS Inc, Somers, NY, USA) as described^38^. In brief, survival analysis utilised the Log-rank test, while cytokine profile was analysed with planned comparisons. For data generated from the IntelliCage, two between-subjects comparisons were made between different mouse groups that had all received CEFT treatment: 1. TLR2/4^+/+^ sham-infected vs. mice cured of meningitis by CEFT (post-meningitic, “PM”); 2. TLR2/4^−/−^ sham-infected vs. PM mice. Mixed ANOVA based on a general linear model (GLM) was used for repeated measurements. For all tests, statistical significance was indicated by *p* < 0.05. Data values that did not fall within the range of mean ± 2 standard deviations were regarded as outliers and were excluded from analysis^38^.

## Supplementary information

Supplementary Information
