# Supplementary material for: Double deficiency of toll-like receptors 2 and 4 alters long-term neurological sequelae in mice cured of pneumococcal meningitis
Source: Sci Rep. 2019 Nov 7;9:16189. doi: 10.1038/s41598-019-52212-7 (PMC6838097; doi:10.1038/s41598-019-52212-7)
Supplement: Supplementary file 1 — Supplementary Information [file 41598_2019_52212_MOESM1_ESM.pdf]

Double deficiency of toll-like receptors 2 and 4 alters long-term neurological sequelae in mice cured of pneumococcal meningitis

Lay Khoon Too[a, *], Belinda Yau[a], Alan G. Baxter[b], Iain S. McGregor[c], Nicholas H. Hunt[a]

[a] *The University of Sydney, Molecular Immunopathology Unit, Bosch Institute and School of Medical Sciences, University of Sydney, Sydney, New South Wales 2006, Australia*

[b]*Comparative Genomics Centre, James Cook University, Townsville, Queensland 4811, Australia*

[c]*School of Psychology, University of Sydney, Sydney, New South Wales 2006, Australia*

Supplementary Table 1 IntelliCage-based measurements of behavioural phenotypes in TLR2/4-deficient C57BL/6J post-meningitic (PM) mice conducted in R1: summary of statistical analysis.

**Test module**	**Investigated parameters**	**Between-subjects comparisons Uninfected vs. PM**							
		**WT**				**GKO**			
		**Between-subjects (BS) comparisons**		**Within-subjects (WS) comparisons**		**Between-subjects (BS) comparisons**		**Within-subjects (WS) comparisons**	
		$\mathbf{F}_{(dfn, dfd)}$	***p***	$\mathbf{F}_{(dfn, dfd)}$	***p***	$\mathbf{F}_{(dfn, dfd)}$	***p***	$\mathbf{F}_{(dfn, dfd)}$	***p***
**a** Free adaptation – Light phase	Cumulative corner visits	$43.06_{(1, 28)}$	**< 0.001**↓	$51.30_{(1.44, 40.37)}$	**< 0.001**↓	$130.25_{(1, 24)}$	**< 0.001**↓	$95.30_{(1.44, 34.66)}$	**< 0.001**↓
	Cumulative corner visits with nosepokes	$91.83_{(1, 24)}$	**< 0.001**↓	$72.00_{(1.24, 29.84)}$	**< 0.001**↓	$48.33_{(1, 27)}$	**< 0.001**↓	$67.52_{(1.48, 39.89)}$	**< 0.001**↓
	Cumulative corner visits with licks	$37.52_{(1, 26)}$	**< 0.001**↓	$32.39_{(1.66, 48.12)}$	**< 0.001**↓	$32.18_{(1, 26)}$	**< 0.001**↓	$35.99_{(1.23, 31.85)}$	**< 0.001**↓
**b** Free adaptation –	Cumulative corner visits	$0.38_{(1, 28)}$	0.545	$0.88_{(1.24, 34.60)}$	0.376	$2.54_{(1, 26)}$	0.123	$2.67_{(1.32, 34.43)}$	0.102

Dark phase	Cumulative corner visits with nosepokes	$5.04_{(1, 29)}$	**0.033**↓	$5.73_{(1.22, 35.41)}$	**0.017**↓	$16.55_{(1, 26)}$	**< 0.001**↓	$10.77_{(1.33, 34.62)}$	**0.001**↓
	Cumulative corner visits with licks	$18.11_{(1, 28)}$	**< 0.001**↓	$13.11_{(1.56, 43.70)}$	**< 0.001**↓	$16.91_{(1, 26)}$	**< 0.001**↓	$9.02_{(1.55, 40.33)}$	**< 0.001**↓
**c** Adaptation period – light phase	Frequency of corner visits	$76.51_{(1, 23)}$	**< 0.001**↓	$11.58_{(1.60, 36.75)}$	**< 0.001**↓	$176.15_{(1, 21)}$	**< 0.001**↓	$7.13_{(1.58, 33.11)}$	**0.005**↓
	Frequency of corner visits with nosepokes	$51.71_{(1, 25)}$	**< 0.001**↓	$13.43_{(1.40, 34.90)}$	**< 0.001**↓	$139.40_{(1, 22)}$	**< 0.001**↓	$15.41_{(2, 44)}$	**< 0.001**↓
	Frequency of corner visits with licks	$23.41_{(1, 26)}$	**< 0.001**↓	$4.67_{(1, 26)}$	**0.040**↓	$119.14_{(1, 22)}$	**< 0.001**↓	$0.12_{(1, 22)}$	0.731
**d** Adaptation period – dark phase	Frequency of corner visits	$0.79_{(1, 26)}$	0.382	$0.32_{(1.49, 38.77)}$	0.674	$1.38_{(1, 25)}$	0.252	$7.87_{(1.38, 34.61)}$	0.004
	Frequency of corner visits with nosepokes	$1.35_{(1, 26)}$	0.255	$2.46_{(1.33, 34.55)}$	0.117	$1.92_{(1, 24)}$	0.178	$13.25_{(1.39, 33.35)}$	< 0.001
	Frequency of corner visits with licks	$0.03_{(1, 26)}$	0.868	$7.39_{(1.43, 37.29)}$	0.005	$2.08_{(1, 21)}$	0.164	$8.68_{(2, 42)}$	0.001

↑ larger values in PM group

↓ smaller values in PM group

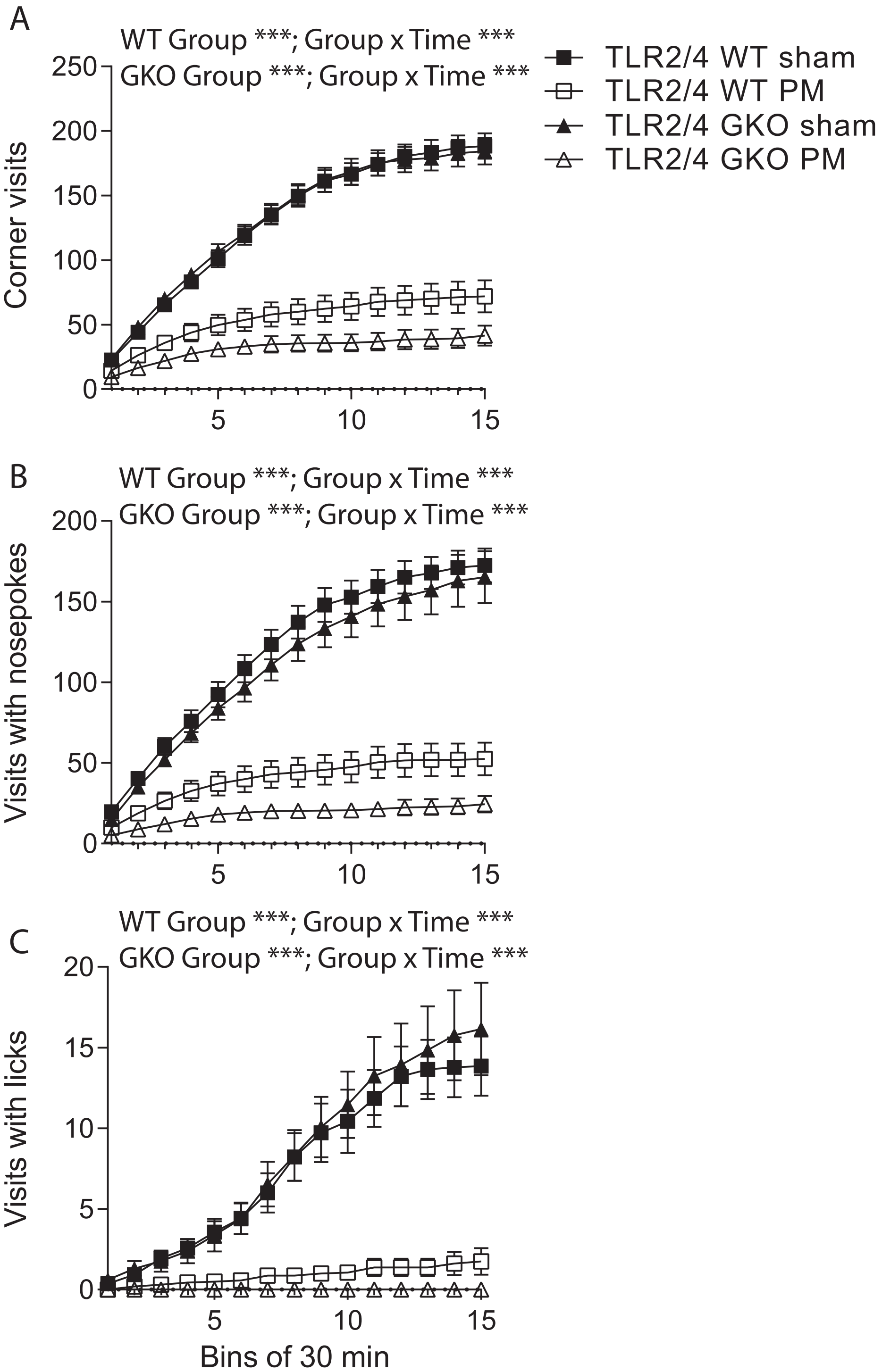

Suppl. Fig. 1: Diurnal exploratory behaviours of mice in early free adaptation in R2. Accumulations of frequency of corner visit (A), of corner visit with nosepokes (B), and of corner visits with water bottle licks (C) are plotted in fifteen 30-min time bins to demonstrate mouse exploratory activities over the initial 5 h of the free adaptation period commencing in the light phase in the re-testing protocol (R2). Total n = 12 – 14 per sham group or n = 15 – 17 per meningitis group. ***$p < 0.001$, mixed ANOVA by GLM repeated measures. Note statistical analysis on cumulative 10-min time bins collected over a 5 h period. A larger time bin (30 min vs. 10 min) is used for graphic clarity. (Abbreviations: GKO = gene knockout, WT = wild-type).

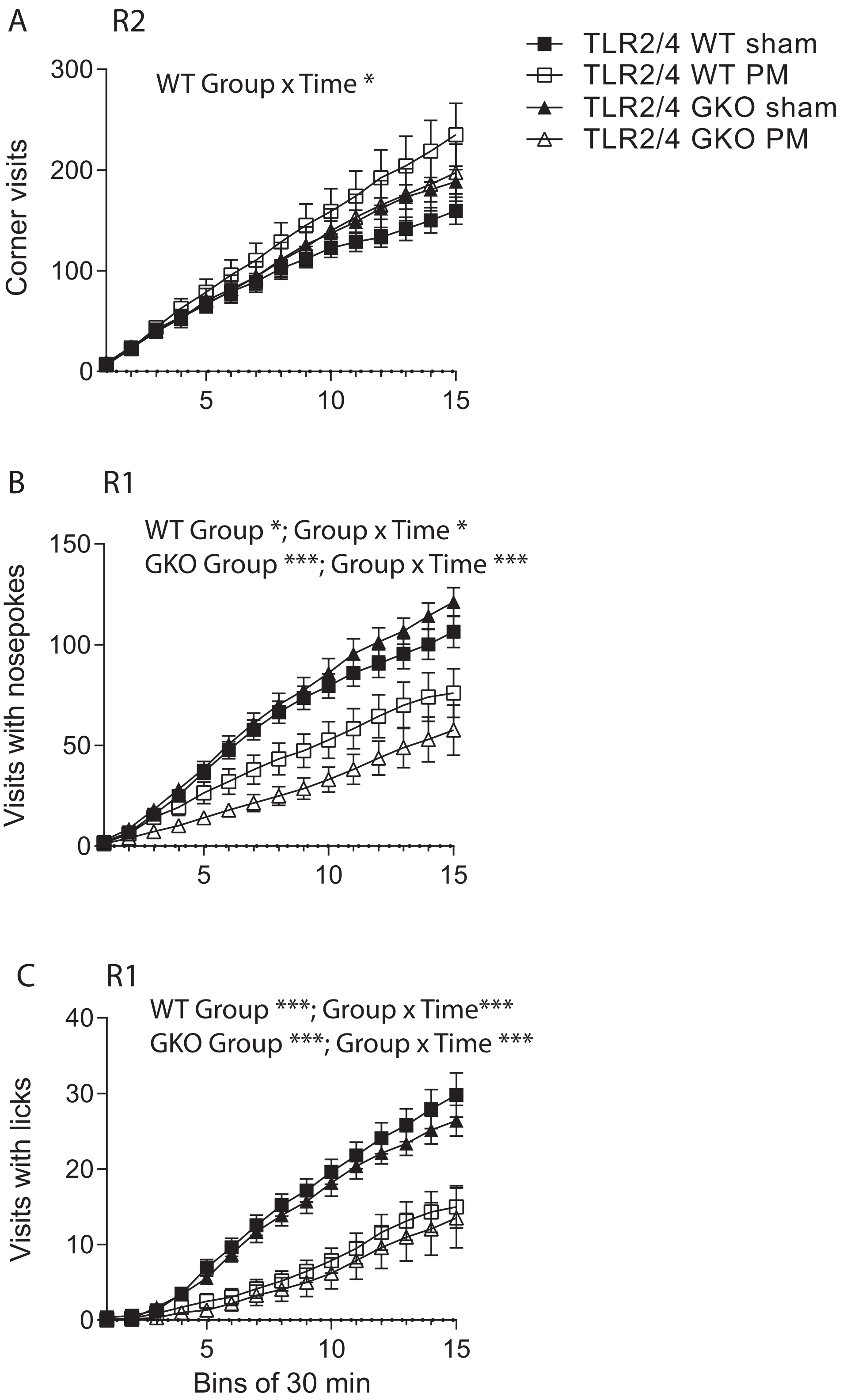

Suppl. Fig. 2: Nocturnal exploratory activities of mice during early dark-phase free adaptation. Accumulations of frequency of corner visit in R2 (A), of corner visit with nosepokes in R1 (C), and of corner visits with water bottle licks in R1 (D) are plotted in fifteen 30-min time bins to demonstrate mouse exploratory activities over the initial 5 h dark cycle of free adaptation period. Total n as indicated in Fig. 3. *$p < 0.05$, ***$p < 0.001$, mixed ANOVA by GLM repeated measures. Note statistical analysis on cumulative 10-min time bins collected over a 5 h period. A larger time bin (30 min vs. 10 min) is used for graphic clarity. (Abbreviations: GKO = gene knockout. WT = wild-type).

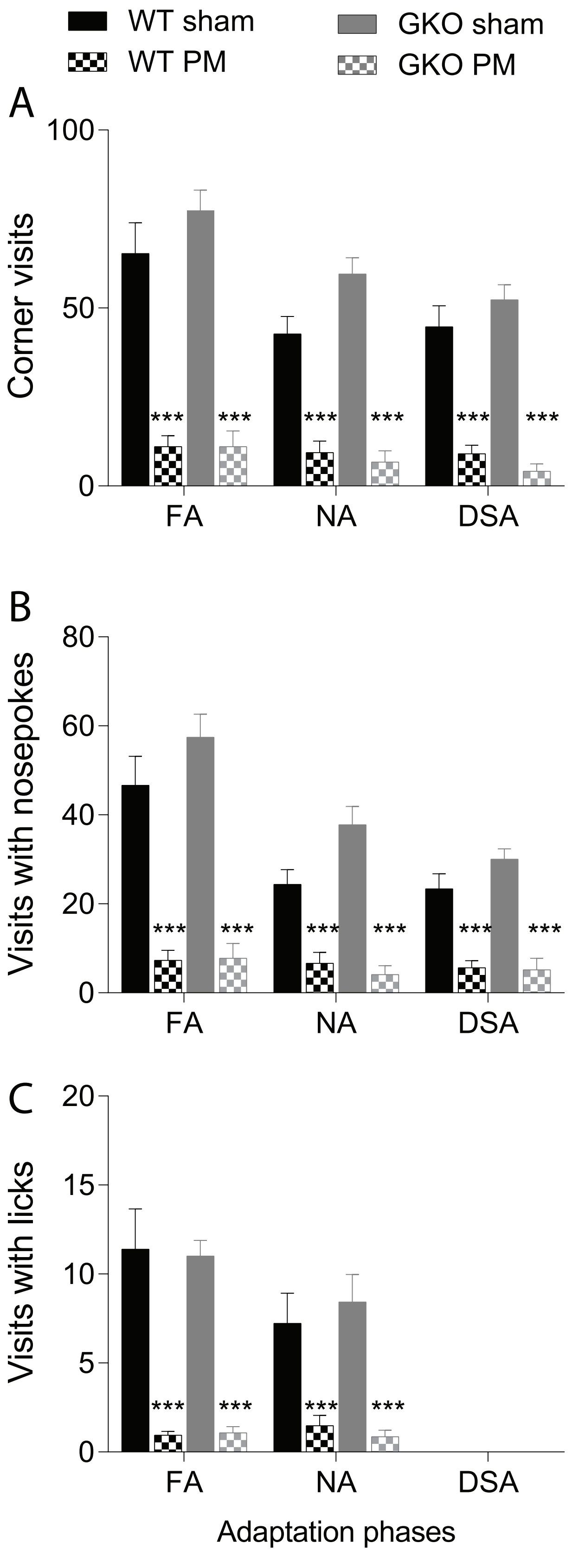

Suppl. Fig. 3: Diurnal exploratory activities of mice during adaptation phases in R2. The frequency of diurnal corner visits (A), of visits with nosepokes (B), and of visits with licks (C) in each two-day free adaptation (FA), nosepoke adaptation (NA) and drinking session adaptation (DSA) was summed and presented. Total n as shown in Fig. 3. ***p < 0.001, group effect as analysed by Fisher's LSD. WT = wild-type; GKO = gene knockout.

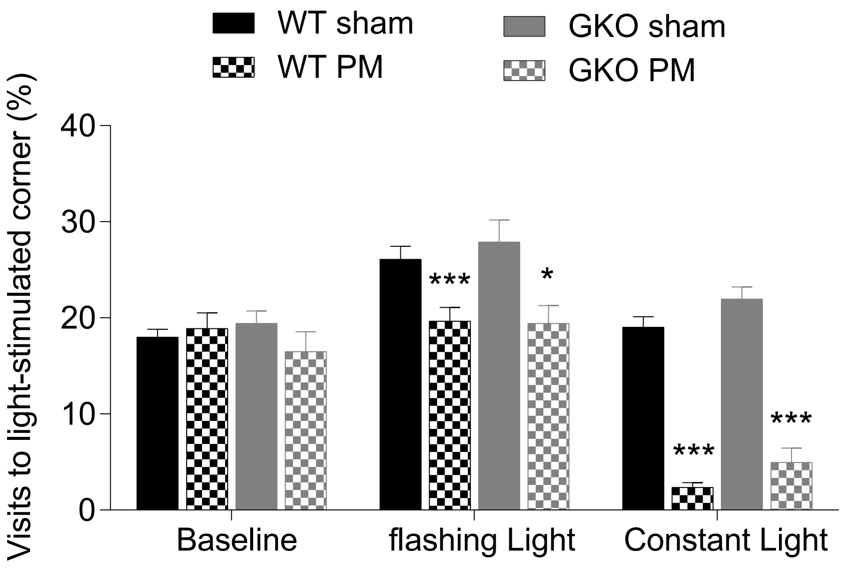

Suppl. Fig. 4: TLR2/4 signalling did not influence the altered light responses in post-meningitic mice (PM). All data are from the re-testing protocol (R2). The percentage of visits to the light-stimulated corner are shown. The light stimulus was changed from no stimulation (baseline) to 5s flashing RBG light upon a corner visit on the first test day, to a constantly-lit RBG on the next day. Total n as shown in Fig. 3. *p < 0.05, ***p < 0.001, group effects as analysed by GLM simple contrasts (baseline as reference category). WT = wild-type; GKO = gene knockout; sham = sham-inoculated.

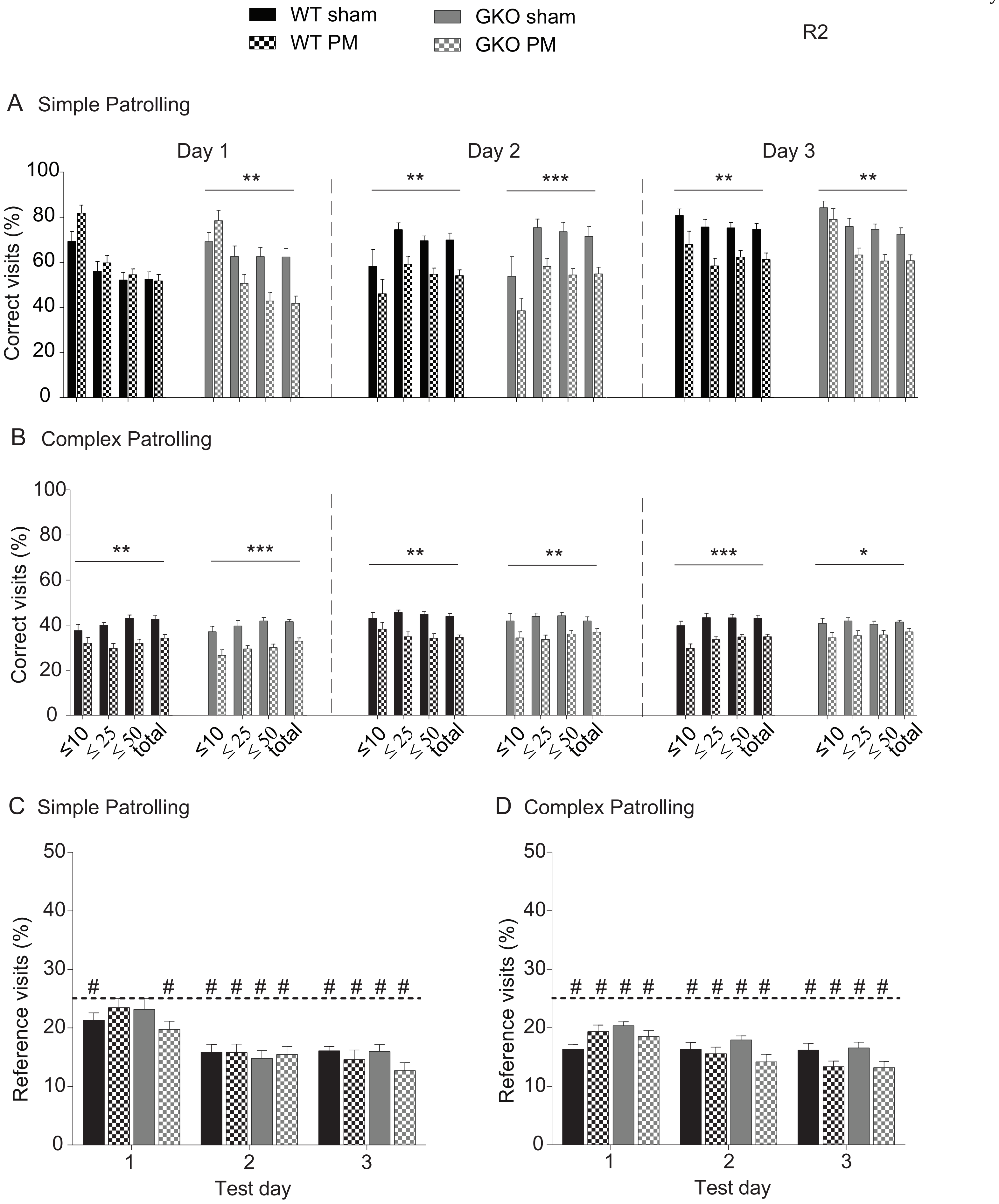

Suppl. Fig. 5: TLR2/4 deficiency did not alter long-term learning performance in post-meningitic mice (PM) subjected to patrolling tasks. All data are from the re-testing protocol (R2). The percentage of correct visits was measured in (A) simple and (B) complex patrolling tasks. The percentage of visits to the non-rewarding reference corners during both (C) simple and (D) complex patrolling tasks also was determined. Total n as shown in Fig. 3. *p < 0.05, ***p < 0.001, group effect on each test day as analysed by Fisher's LSD. "#" indicates percentage value significantly below 25% chance level as analysed by one-sample t test. WT = wild-type; GKO = gene knockout; sham = sham-inoculated.

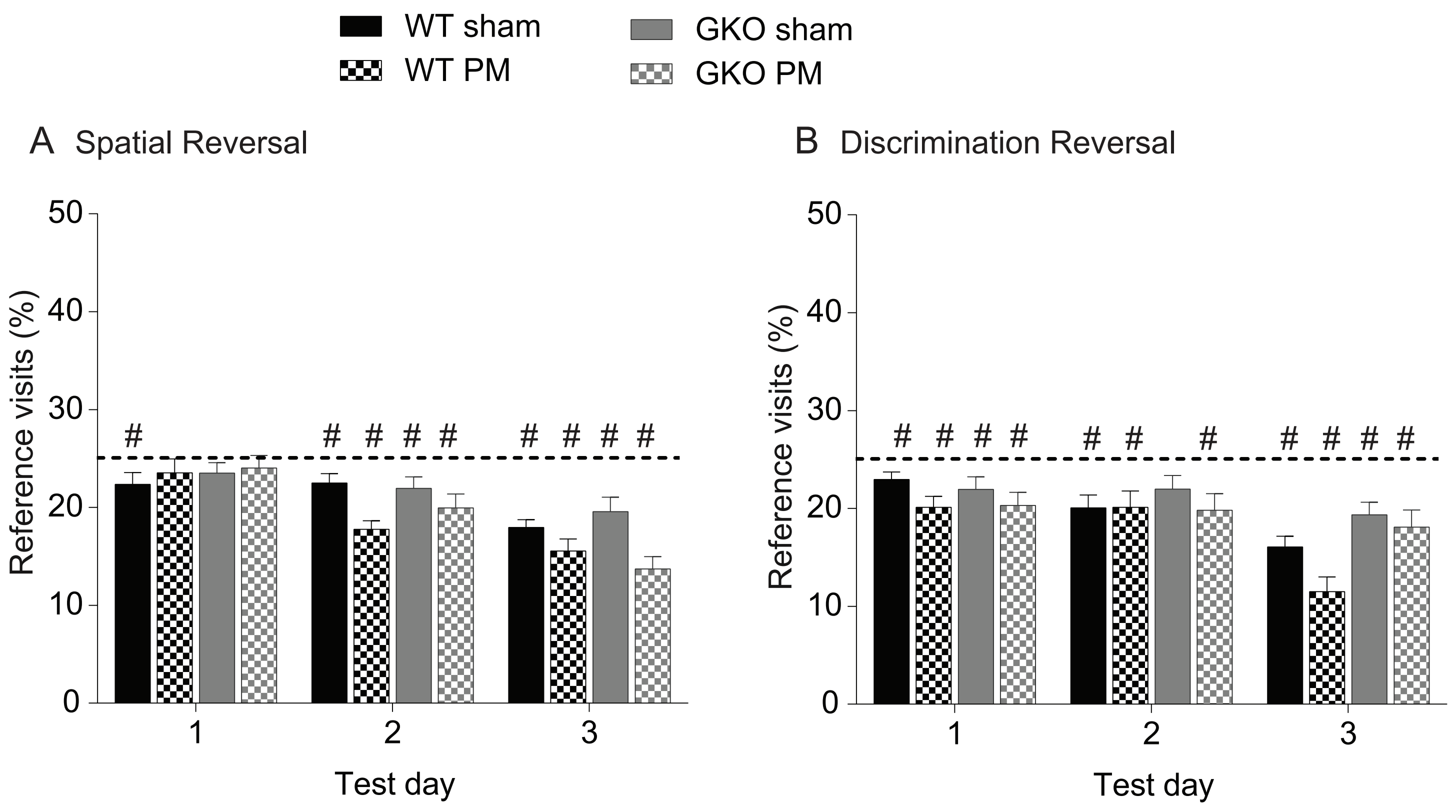

Suppl. Fig. 6: The reference memory of mice in R2. All data are from the re-testing protocol (R2). The percentage of visits to the non-rewarding reference corners during both (A) spatial and (B) discrimination reversals was examined. Total n as shown in Fig. 3. “#” indicates percentage value significantly below 25% chance level as analysed by one-sample t test. WT = wild-type; GKO = gene knockout.
